# Detection of *SHOX* Gene Variations in Patients with Skeletal Abnormalities with or without Short Stature

**DOI:** 10.4274/jcrpe.galenos.2020.2019.0001

**Published:** 2020-11-25

**Authors:** Semra Gürsoy, Filiz Hazan, Ayça Aykut, Özlem Nalbantoğlu, Hüseyin Anıl Korkmaz, Korcan Demir, Behzat Özkan, Özgür Çoğulu

**Affiliations:** 1 University of Health Sciences Turkey, Dr. Behçet Uz Child Disease and Pediatric Surgery Training and Research Hospital, Clinic of Pediatric Genetics, İzmir, Turkey; 2 University of Health Sciences Turkey, Dr. Behçet Uz Child Disease and Pediatric Surgery Training and Research Hospital, Clinic of Medical Genetics, İzmir, Turkey; 3 Ege University Faculty of Medicine, Department of Medical Genetics, İzmir, Turkey; 4 University of Health Sciences Turkey, Dr. Behçet Uz Child Disease and Pediatric Surgery Training and Research Hospital, Clinic of Pediatric Endocrinology, İzmir, Turkey; 5 Manisa City Hospital, Clinic of Pediatric Endocrinology, Manisa, Turkey; 6 Dokuz Eylül University Faculty of Medicine, Department of Pediatric Endocrinology, İzmir, Turkey; 7 Ege University Faculty of Medicine, Department of Pediatric Genetics, İzmir, Turkey

**Keywords:** SHOX gene, short stature, MLPA, sequence analysis, Madelung’s deformity, hearing loss

## Abstract

**Objective:**

*SHOX* gene mutations constitute one of the genetic causes of short stature. The clinical phenotype includes variable degrees of growth impairment, such as Langer mesomelic dysplasia (LMD), Léri-Weill dyschondrosteosis (LWD) or idiopathic short stature (ISS). The aim of this study was to describe the clinical features and molecular results of *SHOX* deficiency in a group of Turkish patients who had skeletal findings with and without short stature.

**Methods:**

Forty-six patients with ISS, disproportionate short stature or skeletal findings without short stature from 35 different families were included. *SHOX* gene analysis was performed using Sanger sequencing and multiplex ligation-dependent probe amplification analysis.

**Results:**

Three different point mutations (two nonsense, one frameshift) and one whole *SHOX* gene deletion were detected in 15 patients from four different families. While 4/15 patients had LMD, the remaining patients had clinical features compatible with LWD. Madelung’s deformity, cubitus valgus, muscular hypertrophy and short forearm were the most common phenotypic features, as well as short stature. Additionally, hearing loss was detected in two patients with LMD.

**Conclusion:**

This study has presented the clinical spectrum and molecular findings of 15 patients with *SHOX* gene mutations or deletions. *SHOX* deficiency should be especially considered in patients who have disproportionate short stature or forearm anomalies with or without short stature. Although most of the patients had partial or whole gene deletions, *SHOX* gene sequencing should be performed in suspected cases. Furthermore, conductive hearing loss may rarely accompany these clinical manifestations.

**What is already known on this topic?**

The phenotypic spectrum of *SHOX* deficiency ranges from Langer mesomelic dysplasia at the severe end of the spectrum to idiopathic short stature at the mild end of the spectrum. Partial or whole *SHOX* gene deletions are usually detected in most of the patients.

**What this study adds?**

*SHOX* deficiency should be especially considered in patients who have disproportionate short stature or forearm anomalies with or without short stature. *SHOX* gene sequencing should also be performed in suspected patients who do not have any deletion/duplication in *SHOX* gene. Additionally, hearing loss might be found in addition to the skeletal and the other clinical features.

## Introduction

Short stature is defined as a height more than two standard deviations (SDs) below the mean for age and sex, compared with national height standards, and affects 2-3% of individuals in the general population. It is a multifactorial disorder as strong genetic and environmental factors are involved (1,2). Several monogenic genetic causes have been identified in short stature and one of these is the short stature homeobox-containing gene *(SHOX)* (3). The *SHOX* gene, which is located in pseudoautosomal region 1 (PAR1) on the short arm of the sex chromosomes Xp22.33 and Yp11.32, escapes X-inactivation. It encodes a nuclear protein which acts as an important transcription factor during limb development (4,5).

The loss of both *SHOX* alleles causes an extreme phenotype of skeletal dysplasia called Langer mesomelic dysplasia (LMD) while *SHOX* haploinsufficiency is associated with a wide spectrum of short stature phenotypes including Turner syndrome, Léri-Weill dyschondrosteosis (LWD) and idiopathic short stature (ISS). LWD is characterized by short stature, mesomelic shortening of the limbs, and characteristic abnormality of the wrists known as Madelung’s deformity. The phenotype can be also highly variable, even within the same family (6,7).

The sensitivity of clinical characteristics in identifying patients with ISS are usually insufficient, especially in younger children in whom skeletal disproportions are not so prominent (1,8). In many cases short stature is also the only clinical manifestation. Mutations or deletions of *SHOX* or *SHOX* regulatory regions have been detected in 75% of the cases with LMD and 60% of the cases with LWD. Additionally mutations of this gene are detected in 2-22% of ISS (9,10). However, partial or complete *SHOX* duplications have been described in a few patients with LWD and ISS (11). Moreover, more than 380 mutations in the coding regions of the gene and mutations in the downstream or upstream enhancer elements have been identified but a clear genotype-phenotype correlation has not been reported (1,12).

The aim of this study was to determine the clinical findings and molecular results of *SHOX* deficiency in a group of Turkish patients with LWD, LMD or ISS.

## Methods

### Patient Selection

Forty-six patients with ISS, disproportionate short stature or skeletal findings without short stature from 35 different families, who were examined at Clinic of Pediatric Genetics, Medical Genetics and Pediatric Endocrinology of Behçet Uz Child Disease and Pediatric Surgery Training and Research Hospital and Department of Medical Genetics of Ege University from Turkey, between June 2014 and July 2019, were included in this study. Data collected included the age, sex, weight, height, body mass index (BMI), and upper segment/lower segment ratios of 15 patients from four different families with *SHOX* gene variation. The clinical and dysmorphic features, anthropometric measurements, skeletal findings including appearance of muscular hypertrophy, cubitus valgus, forearm bowing, Madelung’s deformity, and molecular findings were recorded. A Rappold scoring system was used, which was designed to identify the most appropriate patients for gene testing, and the results were calculated from the medical records of the patients. The score combines three anthropometric variables [arm span/height ratio <96.5% (2 points), sitting height/height ratio >55.5% (2 points) and BMI >50^th^ percentile (4 points)], with five clinical variables [cubitus valgus (2 points), short forearm (3 points), bowing of forearm (3 points), muscular hypertrophy (3 points) and dislocation of the ulna at the elbow (5 points)], each of which represents at least two points in the score system. A score greater than 4 out of a total possible score of 24 is more valuable as a clinical indicator to detect patients with *SHOX* deficiency (1).

ISS is defined as a condition characterized by a height more than two SDs below the mean of the age and sex-matched population in a subject with normal birth size, normal body proportions, normal nutrition, no evidence of chronic disease, no psychiatric or emotional disturbance and no endocrine deficiency (13).

### Statistical Analysis

Statistical analyses were performed using IBM SPSS Statistics for Windows, Version 23.0. (IBM Corp., Armonk, NY, USA). Data was presented with descriptive statistics (median with 25^th^-75^th^ percentiles for continuous variables; frequency and percentage for categorical variables). Student’s t-test or Mann-Whitney U test was used to compare continuous variables, as appropriate. The significance level was accepted as p<0.05 in all statistical analyses. The Local Ethics Committee approved the study (Dr. Behçet Uz Children’s Hospital, Clinical Research Ethics Committee, İzmir; approval number: 2020/01-07), and written informed consent was obtained from all individuals involved.

### Molecular Analysis

### DNA Isolation and Sanger Sequencing

Genomic DNA from peripheral blood lymphocytes of all individuals were extracted with Zinexts MagPurix Blood DNA Extraction Kit (Zinexts Life Science Corp., New Taipei City, Taiwan) using standard procedures. All coding exons and exon-intron boundaries of the *SHOX* gene were amplified by polymerase chain reaction. The sequences were evaluated using SeqScape Software 3 sequencing program (Applied Biosystems SeqScape Software 3, Life Technologies Corporation, 5791 Van Allan Way, Carlsbad, California 92008). “Ensembl.org” database (GRCh38.p12) with ENST00000381578.6 transcript ID of the *SHOX* gene was used to compare individual and reference sequences. All variations were checked using mutation and SNP databases (Human Genome Mutation Database, National Center for Biotechnology Information, ensembl.org). Each variation was confirmed by bidirectional sequencing. Variation descriptions were done according to the nomenclature recommended by the Human Genomic Variation Society. Furthermore, in silico programmes, such as SIFT, PolyPhen 2, and Mutation Taster were used to describe the pathogenity of novel variations in coding exons and exon-intron boundaries.

Multiplex ligation-dependent probe amplification (MLPA) analysis was performed to detect large deletions and duplications using P018 SALSA MLPA Kit (MRC-Holland bv; Willem Schoutenstraat 1 1057 DL, Amsterdam, the Netherlands). The PCR products were analysed by ABI 3500 capillary electrophoresis system (Applied Biosystems 3500/3500xL Genetic Analyzer Life Technologies Corporation, 5791 Van Allan Way, Carlsbad, California 92008) and Coffalyser Software (MRC-Holland, Amsterdam, The Netherlands; http://www.mrc-holland.com). The area under the peak for each amplified fragment was measured and normalized to the peak areas of normal control individuals.

## Results

**Figure 1:**
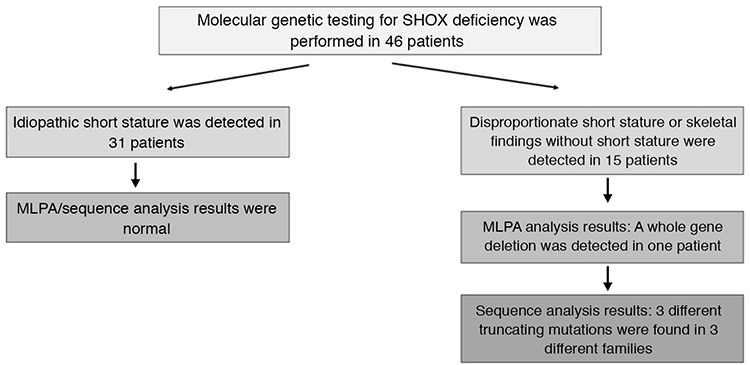
This scheme provides an approach to the study design and molecular results of the patients with/without *SHOX* deficiency

**Figure 2:**
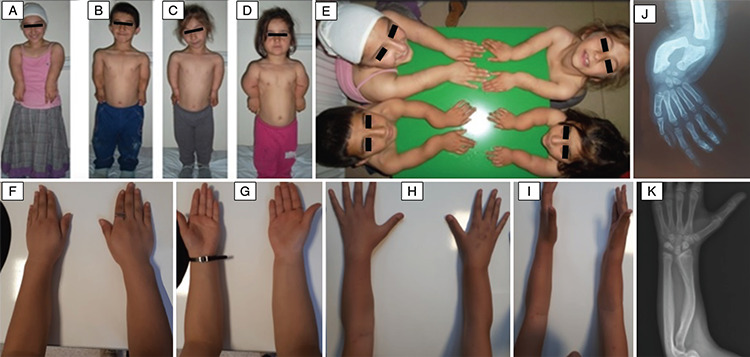
The clinical and radiological findings of the mutation positive patients. A, B, C, D, E) The clinical features of the patients with langer mesomelic dysplasia. F, G) Madelung deformity of patient 15. H, I) Madelung deformity and short forearm of patient 13. J) The direct radiography of patient 2 revealed bowing and shortening of the distal radius, widening of the distal radial-ulnar joint, and triangulation of the distal radial epiphysis, producing an ulnar slant to the articular surface. K) Bowing of forearm, radial bowing and ulnar shaft thickening of patient 13

Forty-six patients from 35 families with idiopathic or disproportional short stature or skeletal findings without short stature were screened for deletions and intragenic mutations of the *SHOX* gene (Figure 1). Mutations in *SHOX* were identified in 15 patients from four different families; three different point mutations and one heterozygous whole *SHOX* gene deletion were detected (Table 1). The skeletal findings (cubitus valgus, Madelung’s deformity, mesomelic shortening, radial bowing) of the mutation positive patients were variable, even within the same family (Figure 2). The median age of the patients with *SHOX* deletion/mutation at referral was 12 years (range, 8-36 years) and five (33%) of them were male. Anthropometric parameters of the cases showed great variation in terms of clinical diagnosis. While the median height SD score (SDS) of the patients with LMD with severe Madelung deformity was -5.5 [range, (-7.1)-(-4.8)], the median height SDS of the patients with LWD was -1.5 [range, (-1.9)-(-1.3)]. The median BMI of the fifteen patients with *SHOX* mutation/deletion was 22.8 (range, 18.3-28.7). The Rappold score was higher than 4 points in all of the patients with *SHOX* deficiency. The other clinical characteristics and molecular findings of the cases are detailed in Table 2. Patients with *SHOX* deficiency also showed significantly higher BMI SDS levels than patients without *SHOX* deficiency [BMI SDS 1.4 (range, 0.3-2.3), vs. -0.68 (range, -1.56-0.92), p<0.05]. Furthermore, there was no significant difference between these two groups, regarding height and height SDS. The comparison of the demographic features of these two groups were shown in Table 3.

In the first family, a frameshift mutation, c.42delG (p.Ser16AlafsTer60), was detected in all family members (five siblings and the parents). The parents had a consanguineous marriage. A 9 year-old boy (patient 2) and his three affected sisters were found to be homozygous for that mutation and were diagnosed with LMD. In contrast, their other brother and the parents were heterozygous for the same mutation, which favored the diagnosis of LWD. Karyotype analysis was also normal in patient 2 (the index patient). On physical examination, disproportionate short stature, short and webbed neck, low hairline, pectus excavatus, bilateral severe Madelung’s deformity with ulnar deviation, camptodactyly of the 3^rd^-4^th^ digits in right hand and fourth digit in left hand were noted in the patients with LMD. Antero-posterior and lateral radiographs demonstrated the bowing and shortening of the distal radius, widening of the distal radial-ulnar joint, and triangulation of the distal radial epiphysis, producing an ulnar slant to the articular surface (Figure 2). Additionally, audiometric test showed that two of them had bilateral conductive hearing loss (patient 1: right 45 dB, left 45 dB; patient 4: right 45 dB, left 65 dB). Patient 5 had only Madelung’s deformity, which was detected by radiological examination. Besides, the mother had short stature (142 cm) and Madelung’s deformity and was diagnosed as LWD. We could not have contact with the father; only his blood samples were analyzed and we obtained related data from his photographs and wife.

In the second family, a heterozygous nonsense mutation, c.631C>T (p.Q211X), was detected in a 7 year-old girl (patient 8) who had the diagnosis of LWD. Her height was 113 cm (-1.63 SDS) and weight was 23 kg (0.03 SDS). Mild muscular hypertrophy, short forearm, and bowing of the tibia were observed. Madelung deformity and cubitus valgus were not obvious. Her parents and two sisters were found to have similar clinical features and the same mutation. Additionally, the parents had cubitus valgus and Madelung deformity.

In the third family, patient 13 and her father had another heterozygous nonsense mutation, c.492G>A (p.W164X). This 12 year-old girl was referred for multiple skeletal findings. Her height was 143 cm (-1.57 SDS) and mesomelic shortening was detected in upper and lower extremities. Madelung’s deformity with pain and restriction of the flexion/extension were observed in the right forearm. On her radiologic examination, bowing of forearm, especially radial bowing, Madelung’s deformity and ulnar shaft thickening were detected (Figure 2). Abdominal ultrasonography revealed right renal ptosis. Her father, who was 157 cm, had only short stature with mild mesomelic shortening.

Whole *SHOX* gene deletion was detected with MLPA analysis in patient 15, the only member of the fourth family to be affected, who was referred for disproportional short stature. The patient was 14 years old and her height was 142 cm (-3.1 SDS). On her physical examination, cubitus valgus, bowing of the forearm, Madelung’s deformity and short forearm were noticeable. Abdominal ultrasonography was normal. Karyotype analysis was 46,XX. The clinical features and molecular tests were also normal in other members of the family.

## Discussion

In the present study *SHOX* gene molecular defects in patients with LMD, LWD and ISS and the phenotype-genotype spectrum of *SHOX* deficiency were evaluated. In the current literature, point mutations and deletions of the *SHOX* gene have been reported in patients with ISS at an estimated prevalence ranging from 2-22% (9,10). Nevertheless, forearm anomalies and short stature with an increased sitting height/height ratio are most likely to be associated with *SHOX* haploinsufficiency (14,15).

The *SHOX* gene belongs to a family of transcriptional regulators and is essential for the development of the skeleton; especially in the growth and maturation of bones in the arms and legs (16). The clinical expression of *SHOX* deficiency is highly variable and the phenotype usually becomes more pronounced with age, and typical manifestations appear over time (17). While LMD, which is a much more severe skeletal dysplasia than LWD, results from biallelic (homozygous or compound heterozygous) *SHOX* pathogenic variants, *SHOX* haploinsufficiency is associated with ISS and LWD (6). In the present study, four patients from the first family had a homozygous *SHOX* gene mutation and severe skeletal findings, whereas the clinical features of other family members, who had heterozygous mutation, were compatible with LWD. In the second family, the parents had more obvious skeletal manifestations than their daughters. Additionally, while the father of patient 13 had only mild short forearm with short stature, the daughter had Madelung’s deformity, radial bowing and ulnar shaft thickening on limb radiographs. Consistent with the literature, the clinical findings of these patients highlight an intrafamilial phenotypic variability.

In the patients presented, short stature, increased upper segment/lower segment ratio, short forearm, bowing of tibia and appearance of muscular hypertrophy were the most common phenotypic features. Three out of four index patients had at least one affected family member. Additionally, in the first and second family, the parents had a consanguineous marriage and both of them had a heterozygous mutation. As reported in the literature, it is not uncommon for patients with *SHOX* haploinsufficiency to have an affected parent (3).

The combination of dyschondrosteosis and hearing loss has been reported in several cases. In 1970 Nassif et al (18,19) described five siblings with dyschondrosteosis and two of the affected patients had a conductive hearing loss with middle ear deformities. The audiogram revealed bilateral conductive hearing loss of approximately 40-50 dB in both of the patients. In 2003, De Leenheer et al (19,20) reported a patient with a diagnosis of LWD who had a deletion in *SHOX* gene. The patient had short stature, mesomelic shortening and Madelung’s deformity with shortening and bowing of the radius and dorsal dislocation of the ulnar head. The audiogram showed that the patient had unilateral 35 dB conductive hearing loss in the left ear. In our study, bilateral conductive hearing loss was detected in two patients with LMD from the first family. Hearing tests were normal in the other affected siblings. On the basis of these findings and earlier evidence, we suggest that conductive hearing loss may be a rare manifestation of *SHOX* deficiency and a hearing evaluation should be performed in these patients.

The most common mutation is a deletion of part or the entire *SHOX* locus (i.e., 80-90% of cases), whereas point mutations appear to be less frequent (10-20%). The *SHOX* protein contains three characteristic domains: a homeodomain, an SH3 binding domain and an OAR domain. Most of the mutations have been described in the homeobox domain which spans exons 3 and 4. The OAR domain is localized at the C terminal end of the gene and is essential for transactivation (11,16). The homeodomain of the *SHOX* gene mediates several key functions that include nuclear localization, DNA binding and protein-protein interactions. Therefore, mutations located in this region may impair these processes and lead to bone defects (20). Furthermore, the cis-regulatory region of *SHOX* extends to ~ 1Mb of the PAR1 and alterations of these regions may be the cause of the phenotype (21). In the present study, deletion of the whole *SHOX* gene was detected in only one patient. Additionally, three different point mutations (two nonsense, one frameshift) were observed in 14 patients from three different families. Nonsense and frameshift mutations that lead to truncation of the SHOX protein can cause absence of the OAR domain at the C-terminal end, resulting in lack of transactivating function. In our study group, the first family had a heterozygous or homozygous frameshift mutation in exon 2, c.42delG (p.Ser16AlafsTer60), which caused the lack of the HD, SH3 and OAR domains. While the second family had a nonsense mutation, c.631C>T (p.Q211X), which was located in exon 5, another nonsense mutation, c.492G>A (p.W164X), which was located in exon 4 and the homeodomain region was detected in the third family. Although the rate of gene deletions is high in *SHOX* deficiency, gene sequencing should be performed in suspected cases. There is also a wide range of phenotypic variations associated with mutations or deletions in the *SHOX* gene. In the current study, point mutations were detected in different exons, but no correlation was found between the severity of phenotype and the underlying *SHOX* pathogenic variant.

### Study Limitation

The major limitation of our study is the relatively small size of the sample.

## Conclusion

In conclusion, the clinical findings and molecular manifestations of four different *SHOX* alterations in four different families are presented. Screening for *SHOX* deficiency should be considered in children with disproportionate short stature or forearm abnormalities with and without short stature. Furthermore, the fact that conductive hearing loss may accompany clinical manifestations should be kept in mind. Genetic diagnosis is essential for the management of the disease and prediction of prognosis. Future studies and identification of further *SHOX* modifier genes will allow better understanding of the phenotype-genotype correlation.

## Figures and Tables

**Table 1:**
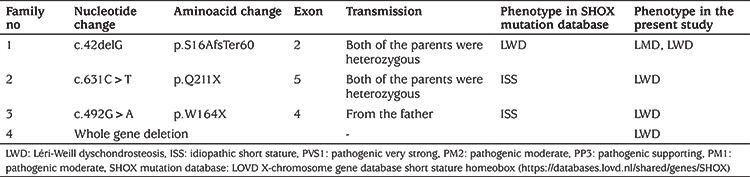
The molecular details of the *SHOX* gene alterations

**Table 2:**
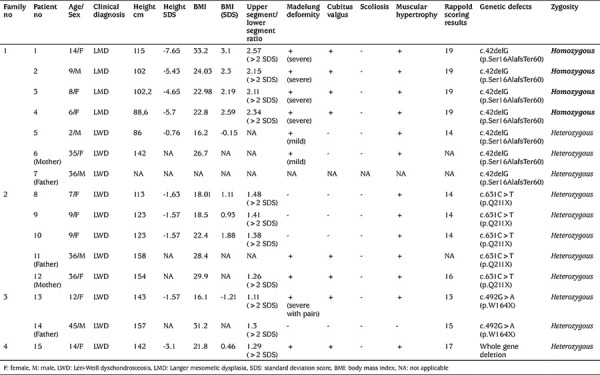
Clinical and molecular findings of the mutation/deletion positive patients

**Table 3:**
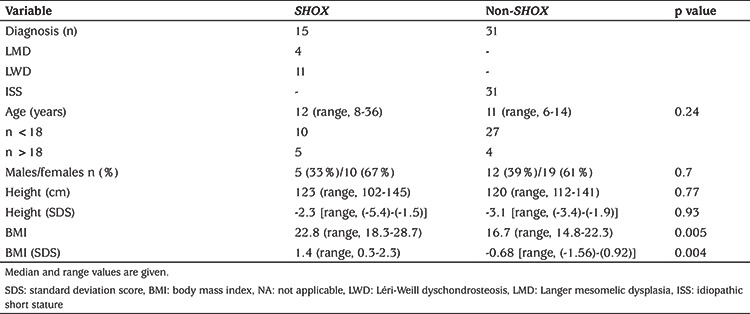
Demographic and anthropometric findings of the patients with/without *SHOX* deficiency
